# TLR3 Activation of Intratumoral CD103^+^ Dendritic Cells Modifies the Tumor Infiltrate Conferring Anti-tumor Immunity

**DOI:** 10.3389/fimmu.2019.00503

**Published:** 2019-03-20

**Authors:** Emiliano Roselli, Paula Araya, Nicolás Gonzalo Núñez, Gerardo Gatti, Francesca Graziano, Christine Sedlik, Philippe Benaroch, Eliane Piaggio, Mariana Maccioni

**Affiliations:** ^1^Department of Clinical Biochemistry, Faculty of Chemical Sciences, Center for Research in Clinical Biochemistry and Immunology, National University of Cordoba, Cordoba, Argentina; ^2^Institute of Experimental Immunology, University of Zurich, Zurich, Switzerland; ^3^Fundación para el Progreso de la Medicina, Laboratorio de Investigación en Cáncer, Cordoba, Argentina; ^4^INSERM U932, Institut Curie, Paris, France

**Keywords:** TLR3, CD103^+^ cDC1, cancer immunotherapy, dsRNA mimetics, tumor-infiltrate

## Abstract

An important challenge in cancer immunotherapy is to expand the number of patients that benefit from immune checkpoint inhibitors (CI), a fact that has been related to the pre-existence of an efficient anti-tumor immune response. Different strategies are being proposed to promote tumor immunity and to be used in combined therapies with CI. Recently, we reported that intratumoral administration of naked poly A:U, a dsRNA mimetic empirically used in early clinical trials with some success, delays tumor growth and prolongs mice survival in several murine cancer models. Here, we show that CD103^+^ cDC1 and, to a much lesser extent CD11b^+^ cDC2, are the only populations expressing TLR3 at the tumor site, and consequently could be potential targets of poly A:U. Upon poly A:U administration these cells become activated and elicit profound changes in the composition of the tumor immune infiltrate, switching the immune suppressive tumor environment to anti-tumor immunity. The sole administration of naked poly A:U promotes striking changes within the lymphoid compartment, with all the anti-tumoral parameters being enhanced: a higher frequency of CD8^+^ Granzyme B^+^ T cells, (lower Treg/CD8^+^ ratio) and an important expansion of tumor-antigen specific CD8^+^ T cells. Also, PD1/PDL1 showed an increased expression indicating that neutralization of this axis could be exploited in combination with poly A:U. Our results shed new light to promote further assays in this dsRNA mimetic to the clinical field.

## Introduction

Cancer immunotherapy using checkpoint inhibitors (CI) is based on neutralizing powerful inhibitory pathways that keep anti-tumor immunity lethargic and poorly effective. A necessary condition for its success is the requirement of pre-existent cytotoxic T cells (CTLs) specific for tumor antigens that will be unleashed through the administration of these CIs (1). Approximately 60% of patients under checkpoint blockade therapy do not respond, probably due to the existence of an ineffective or absent tumor-specific T cell response (i.e., excluded from the tumor site) or an efficient immunosuppressive tumor microenvironment (2).

Indeed, tumors from non-responder patients are characterized by low CD8^+^ T-cell density in their pre-dose biopsies with no appreciable increase in T-cell proliferation post-treatment (3, 4). Hence, great effort is being put into finding new ways to prime the CTL response against tumor antigens with the aim of designing combined therapies that could boost the anti-tumor immune response while keeping the inhibitory circuits in check.

Conventional dendritic cells (cDCs) are the clear candidates to be targeted and among them, a particular subset of cDCs characterized by the expression of the CD103 and CD8α/CD141 markers in mice and humans, respectively (5). CD8α^+^ and CD103^+^ cDC1 in mouse tumors are extremely sparse, yet remarkably capable CTL stimulators. Their development depends on IRF8, Zbtb46, and Batf3 transcription factors. Moreover, cDC1 can produce key chemokines such as CXCL9/10 that will actively recruit more T cells to the tumor site (6, 7). This unique DC subset plays a critical role in taking up antigens inside the tumor bed, trafficking to the nearest tumor-draining lymph node and performing the cross-priming needed to activate the CTL response and eliminate the tumor (7). Therefore, many attempts to target this particular cDC1 population in combination with CI blockade approaches are in the spotlight.

Interestingly, among many innate immune receptors, Toll like receptor 3 (TLR3) has been shown to be expressed almost exclusively by mouse CD8α^+^ cDCs and CD103^+^ cDCs (8) and human CD141^+^ cDCs (9, 10), at least at mRNA level. Therefore, designing new agonists for TLR3 could open new avenues to specifically target and activate these critical populations.

Polyadenylic-polyuridylic acid (poly A:U) is a double-stranded RNA mimetic that was used empirically in cancer immunotherapy in the early 80's with promising results and non-reported toxic effects (11–14). Interestingly, when naked poly A:U is administered locally at the tumor site, it inhibits tumor growth and prolongs survival in several murine cancer models (15). These important anti-tumor effects rely on type I interferon signaling on the host, since they are completely abolished in IFNAR1^−/−^ mice. We have recently shown *in vivo* production of IFNβ as soon as 6 h after poly A:U intratumoral (i.t.) injection. The intratumoral source of the IFNβ required for the efficacy of the poly A:U treatment is a myeloid population within the tumor, carrying the CD11c and LysM markers (15). Interestingly, poly A:U mainly engages TLR3 and no other cytosolic receptors since the production of IFNβ at the tumor site is completely abolished in mice deficient for UNC93B1 signaling, a molecular chaperone strictly required for the activity of nucleic acid sensors, including TLR3 (15, 16). In this work, we used mice heterozygous for the *Tlr3-EGFP* allele (also known as B6-*Tlr3*^*tm*2*Ciphe*^ and called TLR3-GFP knock-in mice here) to identify TLR3^+^ cells within the tumor that therefore represent potentially the main targets of poly A:U and consequently, the putative source of IFNβ inside the tumor. We show that CD103^+^ cDC1 and, to a much lesser extent CD11b^+^ cDC2, are the only populations expressing TLR3 at the tumor site. Upon poly A:U i.t. administration, these cells become activated, and elicit an important tumor-antigen specific CTL response, even in the absence of co-administered tumor antigen (17). Three doses of poly A:U induced profound changes in the composition of the tumor immune infiltrate, switching the immune suppressive tumor environment to anti-tumor immunity. Our results shed new light to promote further assays in this forgotten dsRNA mimetic in the clinical field.

## Materials and Methods

### Mice

Wild-type (WT) C57BL/6J mice were purchased from Universidad Nacional de La Plata, Argentina and Charles River laboratories, France. Mice were maintained at the animal facility at *Institut Curie*. The mice were maintained at the Animal Resource Facility of the *Centro de Investigaciones en Bioquimica Clinica e Inmunologia* in accordance with the experimental ethics committee guideline (*CICUAL*).

### Construction of TLR3-EGFP Knock-in Mice

A 6.27 kb genomic fragment encompassing exons 5 to 7 of the Tlr3 gene was isolated from a BAC clone of B6 origin (clone n° RP23-420M9; http://www.lifesciences.sourcebioscience.com). Using ET recombination, an EGFP-loxP-Cre-neoR-loxP cassette was introduced in the 3′ untranslated region of the Tlr3 gene, with the EGFP sequence fused in frame with that coded by exon 7. The targeting construct was abutted to a cassette coding for the diphtheria toxin fragment A expression cassette, and linearized with Pme1. JM8.F6 C57BL/6N ES cells (18) were electroporated with the targeting vector. After selection in G418, ES cell clones were screened for proper homologous recombination by DNA-PCR. A neomycin-specific probe was used to ensure that adventitious non-homologous recombination events had not occurred in the selected ES clones. Properly recombined ES cells were injected into FVB blastocysts. Germline transmission led to the self-excision of the loxP-Cre-NeoR-loxP cassette in male germinal cells. TLR3-EGFP mice were identified by PCR of tail DNA. The pair of primers: sense 5′- TAAACCATGCACTCTGTTTG−3′ and antisense 5′-GAGTGAATAAACCAGATGTCAA-3′ amplifies a 322 bp band in case of the wild-type Tlr3 allele, whereas the pairs of primers: sense 5′-GAGTACAACTACAACAGCCACA-3′ and antisense 5′-GAGTGAATAAACCAGATGTCAA−3′ amplified a 567 bp band in the case of the Tlr3-EGFP allele. Only mice heterozygous for the *Tlr3-EGFP* allele were used throughout this work since homozygous mice did not respond properly to TLR3 stimulation (Supplemental Figures 1,2).

### Cell Lines

B16-OVA cells were grown in RPMI-1640 containing 10% heat-inactivated FBS (Biowest), 100 IU/ml penicillin, 100 μg/mL streptomycin, 2 mM GlutaMAX (all from Thermo Fisher Scientific). All cell lines were tested as mycoplasma-negative by PCR.

### Isolation of Tumor-Infiltrating Mononuclear Cells

Tumors were harvested and sliced in small fragments that were incubated in RPMI containing DNase at 150 μg/mL (DN25-100MG – Sigma) and Liberase TL at 150 μg/mL (Roche) leaving it in agitation for 20 min at 37°C for enzymatic digestion. Next, tumors were smashed and filtered through a 70 μm cell strainer (BD Cell strainer) and washed with PBS-2%FBS. Afterwards, intratumoral mononuclear cells were purified by density gradient centrifugation using Percoll (GE Healthcare Life Sciences) as specified by the manufacturer.

### Flow Cytometry

Flow cytometric analyses were performed using an LSR Fortessa or FACSCanto II (BD Biosciences). Data were analyzed using FlowJo 10 (Tree Star). DAPI (0.5 mg/mL, Sigma-Aldrich) or a Live/Dead fixable cell stain kit (LIVE/DEAD Fixable Aqua Dead Cell Stain - Thermo Fischer Scientific) was used to exclude dead cells in all experiments. The following antibodies were used for flow cytometry: anti-CD45.2 (clone 104), anti-CD103 (2E7), anti-MHC class II (MHCII) IA/IE (M5/114.15.2), anti-F4/80 (BM8), anti-Ly6C (HK1.4), anti-CD8α (53-6.7), anti-CD206/MMR (C068C2), anti-CD86 (GL1), anti-CD24 (M1/69), anti-CD11c (N418), anti-CD11b (M1/70), anti-CCR2 (SA203G11) anti-CD19 (6D5), anti-TCRβ (H57-597), anti-NKp46 (29A1.4), anti-PDL1 (MIH5), anti-PD1 (29F.1A12), anti-KLRG1 (2F1), anti-CD25 (PC61), anti-Granzyme B (GB11), anti-CD4 (RM4-5). CD8^+^ T cells specific for the OVA peptide SIINFEKL were detected by the iTAg Tetramer/PE - H-2 Kb OVA (SIINFEKL) (TB-5001-1). Refer to Supplemental Table 1 for further information.

#### Intracellular Flow Cytometry

For cytokine staining within myeloid cells, cell suspension from tumors were cultured for 3 h at 37°C in the presence of Brefeldin A (catalog 555029; BD Biosciences) and/or Monensin (catalog 554724; BD Biosciences). After staining for surface markers cells were fixed, permeabilized with Cytofix/Cytoperm (catalog 554722; BD Biosciences), and stained with anti-IL10 (JES5-16E3) for cytokine or anti-Foxp3 (FJK-16s) for Treg identification.

### Experimental Design

Tumor cell lines were established in syngeneic hosts by subcutaneous injection of 5 × 10^5^ B16-OVA cells in 100 μL sterile PBS into the right flank. Tumor development was monitored every other day, with tumor volume determined by the formula (L × W × W)/2 where L is tumor length and W tumor width. When tumors reached a measurable size (~day 7 post-inoculation), a group of mice was intratumorally treated with 100 μL PBS or 100 μg pAU (in 100 μL of PBS) three times every second day. Polyadenylic-polyuridylic acid (poly A:U) was purchased from InvivoGen (catalog ID: tlrl-pau) and handled as specified by the supplier.

### Statistical Analysis

Data handling, analysis, and graphic representation (all shown as mean±SEM, unless specified otherwise) were performed using Prism 7.0 (GraphPad Software). For multiple comparison, one-way or two-way analyses of variance (ANOVA) with Sidak's post-test were performed. For the comparison between two groups, Student's *t*-test was performed. A *p* < 0.05 was considered statistically significant (^*^p < 0.05, ^**^p < 0.01, ^***^p < 0.001, ^****^p < 0.0001). Flow cytometer data were compensated, exported with FlowJo software (version 10.0.8, TreeStar Inc.) and normalized using Cyt MATLAB (version 2017b) followed by unsupervised validated clustering approaches (tSNE, FlowSOM).

## Results

### CD103^+^ cDC1 Are the Main Cells Expressing TLR3 and Key Targets of Poly A:U Inside the Tumor

**Figure 1:**
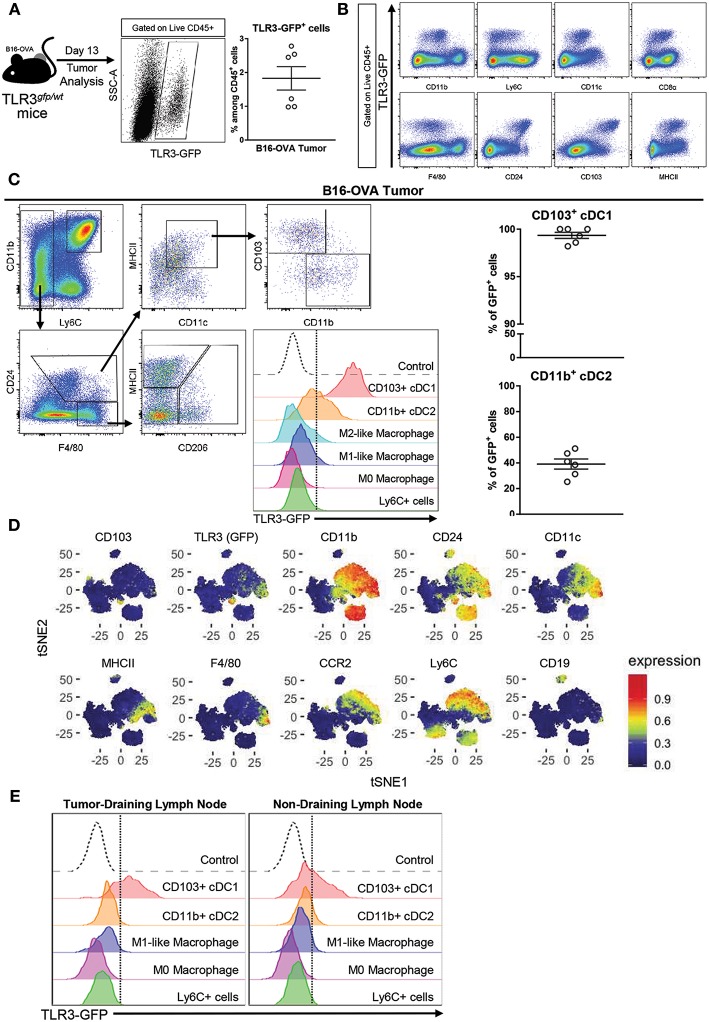
TLR3 is mainly expressed by CD103^+^ cDC1 and a fraction of CD11b^+^ cDC2. **(A)** Expression of TLR3-GFP on tumor-infiltrating leukocytes (CD45^+^ cells) isolated from B16-OVA tumors harvested from TLR3^gfp/wt^ mice at day 13 after tumor cell inoculation. *n* = 6. **(B)** Intratumoral immune cells (Live CD45^+^ cells) from TLR3^gfp/wt^ mice showing expression of TLR3-GFP together with different population markers. **(C)** Gating strategy used to characterize tumor-infiltrating myeloid cells. Expression of TLR3-GFP on different tumor-infiltrating myeloid cells (middle panel). Frequency of CD103^+^ cDC1 and CD11b^+^ cDC2 expressing TLR3-GFP among total cells in each population (right panel). **(D)** tSNE dimensionality reduction showing concatenated flow cytometry data of intratumoral immune cells from TLR3^*gfp*/*wt*^ mice with heat-map showing the distribution of various surface markers on the different clusters. **(E)** Histograms showing TLR3-GFP expression on different myeloid cells present in tumor-draining and non-draining inguinal lymph nodes. Data in **(A,C)** are shown as mean ± SEM.

In order to determine which cell population could be the main target of poly A:U inside the tumor, B16-OVA tumor cells were inoculated in heterozygous TLR3^*gfp*/*wt*^ reporter mice. At day 13 post-inoculation, the mice were sacrificed and tumors were harvested to examine them for GFP expression in tumor-infiltrating immune cells. Roughly 2% of the total CD45^+^ cells infiltrating the tumor were GFP^+^ (Figure 1A) and showed population markers broadly used to identify myeloid cells with the following phenotype CD11b^lo/int^, CD11c^int^, F4/80^lo/int^, CD8α^int^, CD24^hi^, CD103^hi^, MHCII^hi^ (Figure 1B). Following the gating strategy reported to discriminate distinct myeloid populations infiltrating solid tumors (19), cDCs were distinguished from macrophages based on CD24^hi^ and F4/80^lo^ expression, within MHCII^hi^CD11c^hi^ cells. Subsequently, cDCs were found to parse into the two populations already described based on differential expression of CD11b and CD103 (19). We could readily visualize that the totality of the CD103^+^ cDC1 population expressed GFP and had the highest mean fluorescence intensity (MFI) compared to the other myeloid populations. In contrast, GFP expression was detected in ~40% of the CD11b^+^ cDC2, although at significant lower MFI than in CD103^+^ cDC1s (Figure 1C). These findings were supported by reported transcriptomic data that showed that CD8α^+^ cDCs (and some subsets of B cells) were among the most important population expressing TLR3 in mouse spleen (20, 21). These results were verified with an unbiased overview by systematically reducing the flow cytometry data to two dimensions by applying the t-distributed stochastic neighbor embedding (t-SNE) algorithm (22–24) (Figure 1D). By using this unbiased approach we confirmed that, although cells with classical macrophage markers such as F4/80 show some expression of TLR3-GFP, the highest expression of TLR3-GFP was again observed in the CD103^+^ cDC1 Interestingly, similar findings were observed in tumor-draining lymph nodes and non-draining lymph nodes, although in both cases, GFP expression in CD11b^+^ cDC2 was hardly detected (Figure 1E).

These findings indicate that cDCs, and particularly CD103^+^ cDC1 are the main target of poly A:U inside the tumor, and support the hypothesis that they are the main source of IFNβ after poly A:U administration (15).

### Poly A:U Administration at the Tumor Site Exhaustively Modifies the Tumor Immune Infiltrate

**Figure 2:**
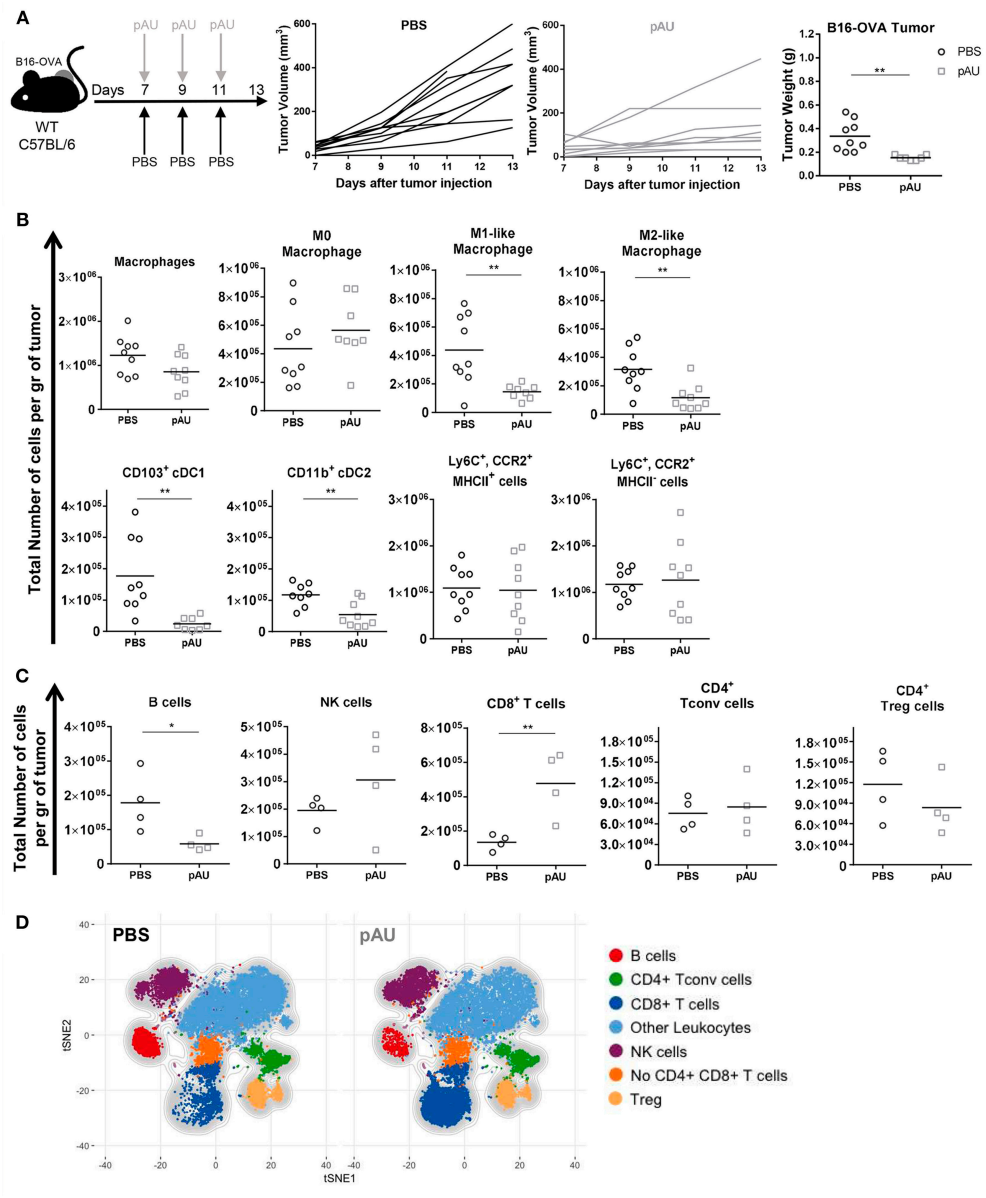
Poly A:U administration at the tumor site exhaustively modifies the tumor immune infiltrate. **(A)** WT C57BL/6 mice bearing B16-OVA tumors were intratumorally -treated with either poly A:U (100 μg/mice/dose) or PBS (control) every other day as indicated in the upper scheme. Plot of individual tumor volume of poly A:U and PBS groups. Tumor weight was evaluated at day 13 post-inoculation. **(B)** Total number of intratumoral myeloid cells per gram of tumor (density). **(C)** Total number of intratumoral lymphoid cells per gram of tumor (density). **(D)** tSNE plots showing concatenated flow cytometry data of intratumoral immune cells from mice treated with PBS (control) or poly A:U (pAU) showing the distribution of the lymphoid populations. *Ex vivo* analyses were performed at day 13 post-tumor inoculation. Data in **(A–C)** are shown as mean and pooled over two cohorts with significance determined by unpaired *t*-test. *n* = 9/group. *p < 0.05; **p < 0.01.

As shown previously by our group, repeated poly A:U administrations at the site of the tumor significantly inhibits tumor growth, as seen by the reduced tumor weight at the time of sacrifice of wild-type (WT) C57BL/6 mice (Figure 2A). In order to exhaustively characterize the changes in tumor infiltrate elicited by poly A:U administration, we first dissected the myeloid compartment, using a 12-color flow cytometry panel and a progressive gating strategy (Supplemental Figures 3A,B). Subgating CD45^+^ infiltrating cells by the myeloid-specific marker CD11b and the monocyte marker Ly6C allowed the identification of monocyte-derived cells inside the tumor that specifically express the chemokine receptor CCR2. Interestingly, two populations of monocyte-derived cells could be distinguished according to their expression of MHC class II (MHCII) molecule in approximately equal number and frequency (Figure 2B and Supplemental Figures 3A,B).

Intratumoral cDCs were distinguished from macrophages as indicated above (CD24^hi^F4/80^lo^MHCII^hi^CD11c^hi^) and further divided into CD103^+^ cDC1 and CD11b^+^ cDC2. On the other hand, parsing of the F4/80^hi^CD24^lo^ compartment revealed distinct types of macrophages further delineated by the expression of MHCII and CD206 as M0 (MHCII^neg^CD206^neg^), M1-like (MHCII^hi^CD206^neg^) and M2-like macrophages (MHCII^lo^CD206^hi^) (Supplemental Figure 3A) (25). The co-stimulatory molecule CD86 was also differentially expressed by these three subsets of macrophages (Supplemental Figure 3B). Granulocytes (Ly6C^int^CCR2^lo^CD24^hi^) were identified but not analyzed.

In accordance with previous reports (26), the different identified myeloid subsets composed almost 50% of tumor-infiltrating leukocytes (CD45^+^), the monocyte-derived cells (28%) and macrophages (14%) being the most abundant ones (Supplemental Figures 4A,C, 5B). The administration of poly A:U did not impact significantly on the density of monocyte-derived cells nor M0 macrophages but decreased the density of M1-like and M2-like macrophages as well as both subsets of cDCs within the tumor (Figure 2B). We then dissected the lymphoid compartment characterizing B (CD19^+^), NK (NKp46^+^), and T (TCRβ^+^NKp46^neg^) cells by using a 14-color flow cytometry panel (Supplemental Figure 3A). The major impact of poly A:U administration was an increase in the density of CD8^+^ T cells whose frequency varied from 8 to 22% among CD45^+^ cells (Figure 2C) and (Supplemental Figures 4B,C). No statistically significant changes were observed on the density nor frequency of Tconv cells, Treg cells, and NK cells (Figure 2C and Supplemental Figures 4B,C). Unexpectedly, tumor infiltrating B cells show a dramatic 75% reduction in their absolute numbers. Applying tSNE algorithms revealed the lymphocytic compartment inside the tumor microenvironment: seven main clusters were identified which confirmed our supervised characterization (Figure 2D and Supplemental Figures 5A–C).

Therefore, recognition of poly A:U by TLR3-expressing CD103^+^ cDC1 generates extensive changes in the number and frequency of many immune cell populations inside the tumor, with an important increase in infiltrating CD8^+^ T cell numbers, which probably impacts the ability of the immune system to control tumor growth.

### Poly A:U Administration at the Tumor Site Reduces the Number of IL10-Producing M2-Like Macrophages, Increases the Number of TNF^+^ Monocyte-Derived Cells and Promotes the Maturation of cDCs

**Figure 3:**
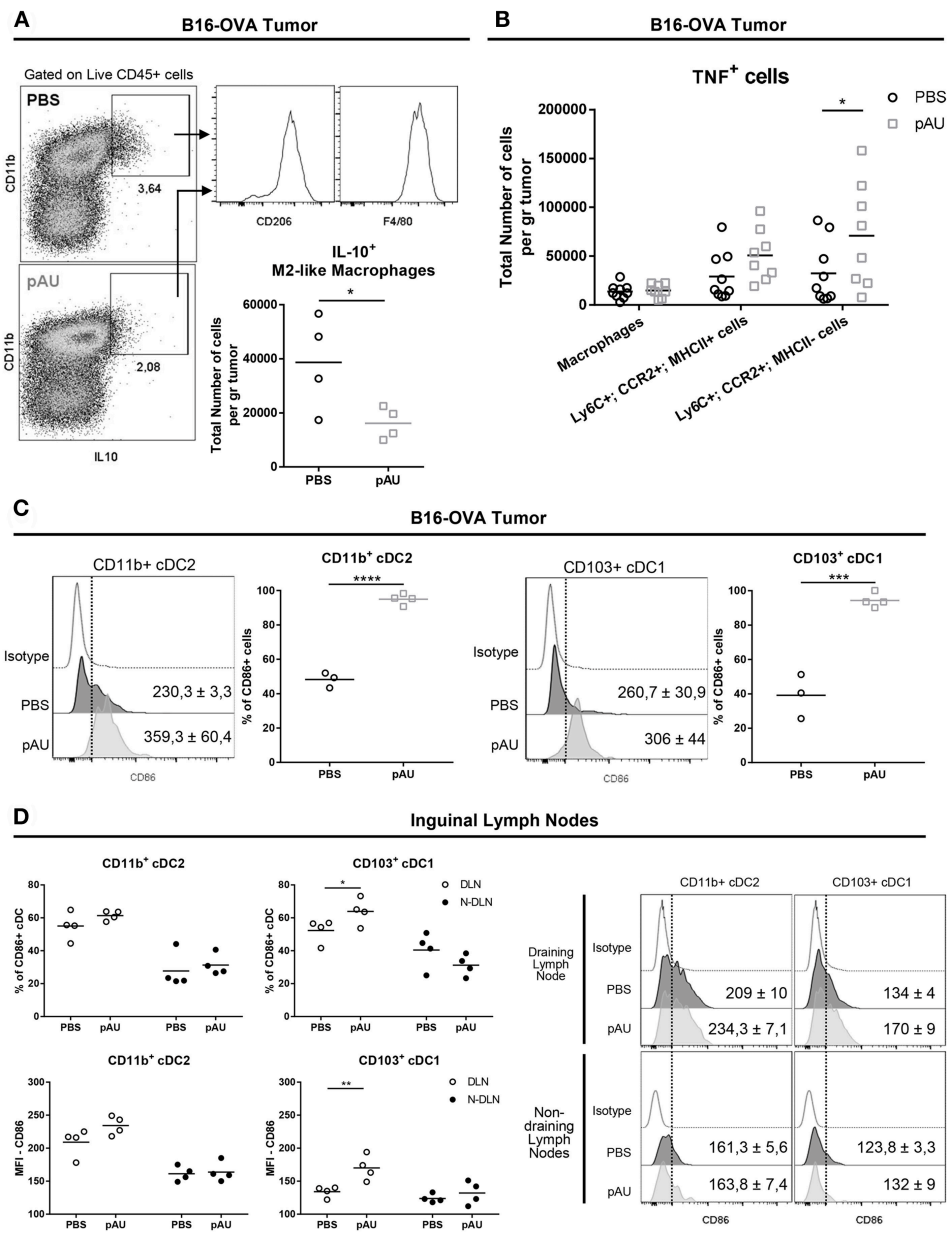
Poly A:U administration at the tumor site reduces the number of IL10-producing M2-like macrophages, increases intratumoral TNF, and promotes maturation/activation of cDCs. **(A)** Representative dot-plots displaying IL10 expression by M2-like macrophages (Ly6C^−^CD11b^+^F4/80^+^CD206^+^). Total number of M2-like macrophages positive for IL10. **(B)** Total number of TNF^+^ cells infiltrating B16-OVA tumors from poly A:U-treated (pAU) and control (PBS) groups. **(C)** Frequency of CD86^+^ cells among intratumoral CD11b^+^ cDC2 and CD103^+^ cDC1 from mice treated with poly A:U (pAU) or control (PBS). Shown are representative histograms for each condition with mean^MFI^±SEM. **(D)** Frequency of CD86^+^ cells among CD11b^+^ cDC2 and CD103^+^ cDC1 present in both tumor-draining lymph nodes (DLN) and non-draining lymph nodes (N-DLN) from mice treated with poly A:U (pAU) or control (PBS). MFI for CD86 in CD11b^+^ cDC2 and CD103^+^ cDC1 among the positive population for this marker. Shown are representative histograms for each condition with mean^MFI^±SEM. *Ex vivo* analyses were performed at day 13 post-tumor inoculation from WT C57BL/6 mice. Data are shown as mean with significance determined by unpaired *t*-test. *n* = 4–5/group. ^*^p < 0.05; ^**^p < 0.01; ^***^p < 0.001; ^****^p < 0.0001.

In order to investigate the impact of poly A:U on the production of the immunosuppressive cytokine IL10 among intratumoral myeloid cells, we looked for the intracellular expression of IL10 in *ex vivo* cells that were not stimulated with PMA/ionomycin, in order to exclude lymphoid sources of this cytokine. Under this condition, we found that the main source of intratumoral IL10 were CD11b^+^F4/80^+^CD206^+^ cells, compatible with M2-like macrophages (Figure 3A). Interestingly, poly A:U decreased by half the density of M2-like macrophages producing IL10 inside the tumor bed (Figure 3A), and at the same time expanded the number of monocyte-derived cells producing TNF, particularly among those that did not express MHCII (Figure 3B).

Despite the reduced total number of cDCs (CD11b^+^ and CD103^+^) found in tumor infiltrates from poly A:U-treated animals, greater proportions of them were activated as judged by CD86 expression level (Figure 3C). To evaluate whether the reduced numbers of cDCs in the tumor infiltrate was a consequence of a reduced/increased trafficking of cDCs to the lymph node, we analyzed the cDC composition of the tumor-draining lymph nodes. Although the number of cDCs in lymph nodes from treated animals was not modified compared to controls (data not shown), a higher frequency of CD103^+^ cDC1 (but not of CD11b^+^ cDC2) expressing the activation/maturation marker CD86 was observed (Figure 3D).

These results indicate that, besides predictable cDC maturation after poly A:U engagement of TLR3, dynamic and still unraveled processes take place that will end up shaping the immune infiltrate, diminishing IL10-producing macrophages and increasing intratumoral TNF among other changes.

### Poly A:U Administration at the Tumor Site Modifies the Overall Distribution of the T Cell Compartment, Favoring Tumor-Specific Immunity

**Figure 4:**
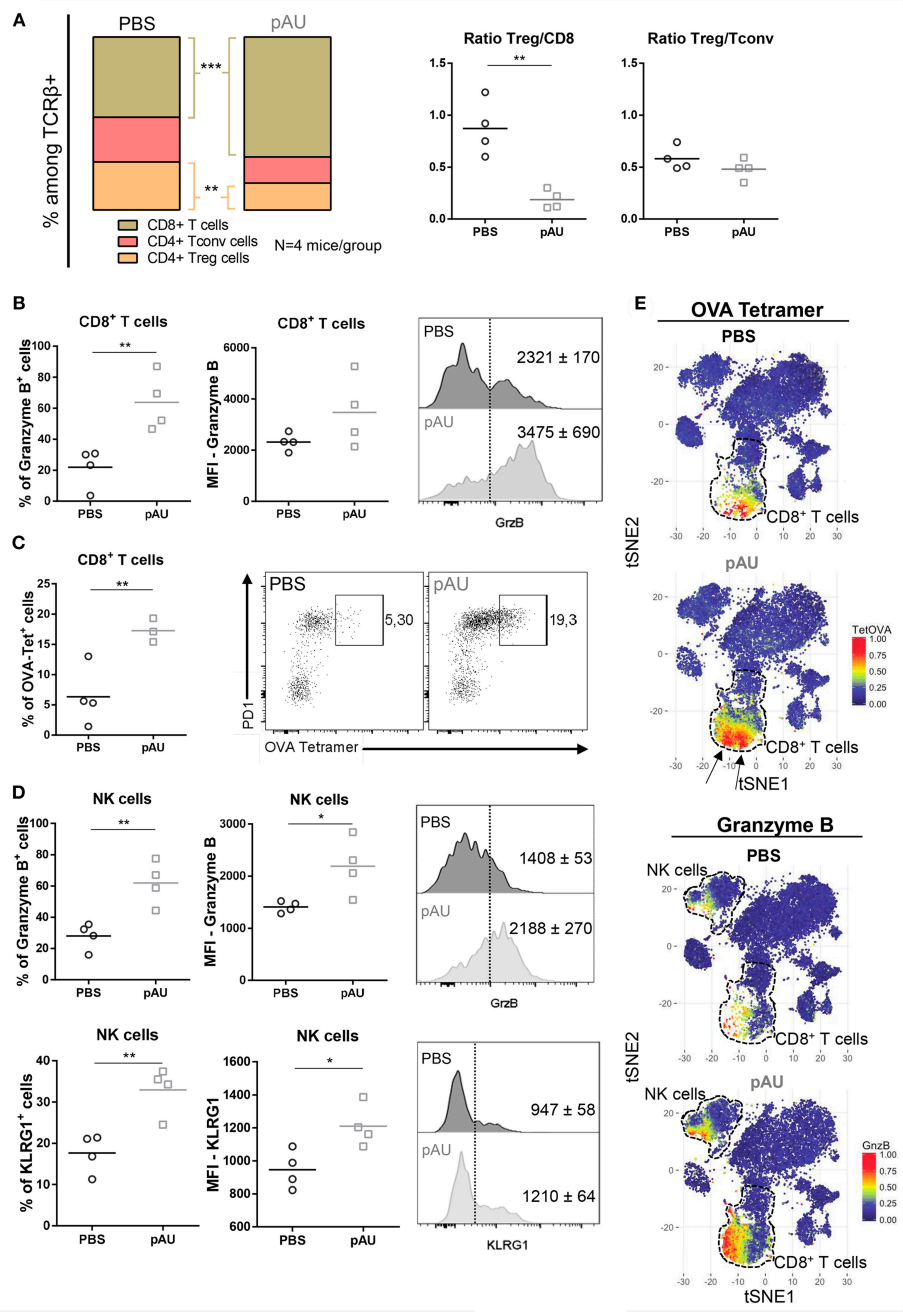
Treatment with poly A:U modifies the T cell compartment, favoring a tumor-specific immune response. **(A)** Frequency of CD8^+^ T cells, CD4^+^ Tconv cells and CD4^+^ Treg cells among intratumoral TCRβ^+^ cells. Intratumoral Treg:CD8^+^ ratio and Treg:Tconv ratio obtained from tumors treated with PBS (control) or poly A:U (pAU) calculated using total number of cells per gram of tumor. **(B)** Frequency of granzyme B^+^ cells among intratumoral CD8^+^ T cells. MFI for granzyme B in intratumoral CD8^+^ T cells among the positive population for this marker. Shown are representative histograms for each condition with mean^MFI^±SEM. **(C)** Frequency of OVA-tetramer^+^ cells among intratumoral CD8^+^ T cells. Shown are representative dot-plots for each condition showing OVA-tetramer^+^ cells expressing PD1. **(D)** Frequency of granzyme B^+^/KRLG1^+^ cells among intratumoral NK cells. MFI for granzyme B/KLRG1 in intratumoral NK cells among the positive population for this marker. Shown are representative histograms for each condition with mean^MFI^±SEM. **(E)** tSNE dimensionality reduction showing concatenated flow cytometry data of intratumoral immune cells from mice treated with PBS (control) or poly A:U (pAU) with heat-map showing the distribution of OVA-tetramer^+^ cells indicated by arrows (upper panel) and granzyme B^+^ cells (lower panel). *Ex vivo* analyses were performed at day 13 post-tumor inoculation from WT C57BL/6 mice. Data in **(A–D)** are shown as mean with significance determined by unpaired *t*-test. *n* = 4/group. *p < 0.05; **p < 0.01; ***p < 0.001.

After characterization of the myeloid component of the tumor infiltrate, we dissected the lymphoid compartment. Poly A:U-treated tumors harvested from WT C57BL/6 mice exhibited large modifications in the overall distribution of the various infiltrating T cell populations (Figure 4A). Poly A:U treatment did not modify the Treg/Tconv ratio inside the tumor, but strikingly reduced the Treg/CD8 ratio, a fact that has been associated with a good prognostic outcome (27) (Figure 4A). Poly A:U treatment also substantially increased the percentage of granzyme B^+^ CD8^+^ T cells (Figure 4B). Moreover, these cells expressed higher levels of granzyme B compared to PBS-treated controls (Figure 4B). Looking at the antigen-specific T cell response, we used OVA-tetramer to quantify the proportion of tumor-antigen specific CD8^+^ T cells. Interestingly, the proportion of OVA-tetramer^+^ CD8^+^ T cells increased in frequency and most of these cells appeared activated as judged by their high level of expression of program cell death-1 (PD1) (Figure 4C). Looking at the NK cell compartment, we observed that poly A:U-treatment also resulted in a higher proportion of granzyme B^+^ and KLRG1^+^ NK cells and higher expression levels of these effector molecules (Figure 4D). The latter results obtained by manual gating, were also validated by the unbiased analysis performed by applying the t-SNE algorithm (Figure 4E). tSNE plots revealed not only the increase in the frequency of CD8^+^ T cells in poly A:U-treated tumor infiltrate, but also that the cluster of OVA-tetramer^+^ CD8^+^ T cells encompassed two definite zones within the CD8^+^ T cell area (Figure 4E). Granzyme B expression on the tSNE projection suggested the existence of a population of CD8^+^ T cells specific for OVA, which do not produce granzyme B. The unsupervised analysis also confirmed that NK cells produced greater levels of granzyme B in poly A:U-treated than in non-treated tumors (Figure 4E). Our data so far indicated that the administration of poly A:U alone at the tumor site, was capable of increasing the frequency of granzyme B^+^ CD8^+^ T cells. Even though poly A:U was administered in the absence of exogenously administered OVA as the tumor-antigen, it was capable of amplifying an OVA-specific CTL response.

**Figure 5:**
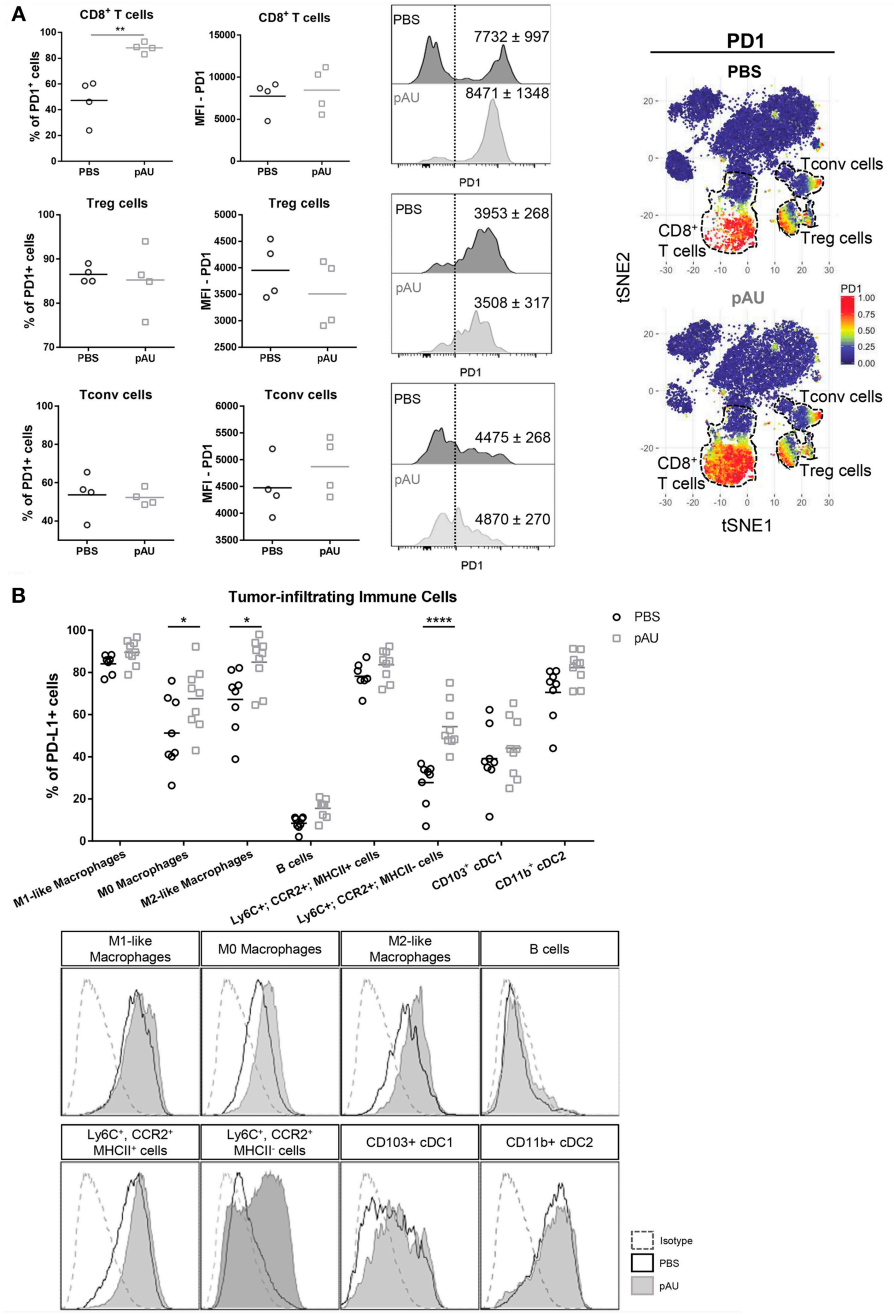
Administration with poly A:U at the tumor bed impacts the PD1/PDL1 axis. **(A)** Frequency of PD1^+^ cells among intratumoral CD8^+^ T cells, CD4^+^ Treg cells and CD4^+^ Tconv cells. MFI for PD1 in CD8^+^ T cells (upper panel), CD4^+^ Treg cells (middle panel), and CD4^+^ Tconv cells (lower panel) among the positive population for this marker. Shown are representative histograms for each condition with mean^MFI^±SEM. tSNE dimensionality reduction showing concatenated flow cytometry data of intratumoral immune cells from mice treated with PBS (control) or poly A:U (pAU) with heat-map showing the distribution of PD1^+^ cells among the CD8^+^ T cells, CD4^+^ Treg cells, and CD4^+^ Tconv cells clusters (dotted lines). **(B)** Frequency of PD-L1^+^ cells within the different subsets of intratumoral immune cells (upper panel). PD-L1 expression in representative histograms for each condition (lower panel). *Ex vivo* analyses were performed at day 13 post-tumor inoculation from WT C57BL/6 mice. Data in **(A,B)** are shown as mean with significance determined by unpaired *t*-test. *n* = 4–9/group. *p < 0.05; **p < 0.01; ****p < 0.0001.

In order to investigate the PD1/PDL1 axis in our model, we evaluated PD1 expression on CD8^+^ T cells, Treg cells and Tconv cells. Our results applying manual gating analysis indicated that the frequency of PD1^+^ CD8^+^ T cells, but not of Treg nor Tconv, was highly increased in poly A:U-treated tumors (Figure 5A). This was corroborated by unsupervised analysis using the t-SNE algorithm: PD1^+^ cells mostly clustered in the Treg, Tconv and CD8^+^ T cell areas. However, there was a striking increase in the size of the CD8^+^ T cell cluster. We then examined PD-L1 expression within the myeloid compartment. Most intratumoral myeloid cells expressed PD-L1 at high levels (Figure 5B), and the frequency of PD-L1^+^ cells was consistently high in every population infiltrating non-treated tumors. Indeed, the frequency of PD-L1^+^ cells was >50% in all the myeloid populations analyzed except for Ly6C^+^CCR2^+^MHCII^neg^ cells. Treatment with poly A:U significantly increased the percentage of M0, M2-like, and Ly6C^+^CCR2^+^MHCII^neg^ cells expressing PD-L1 (Figure 5B), supporting the hypothesis that combining poly A:U with anti PD1/PDL1 inhibitors could potentiate anti-tumor immunity.

## Discussion

Nowadays, the major concern regarding CI blockade immunotherapy is how to enlarge the number of responder patients. Several combinatorial approaches have been explored, mostly in preclinical settings, with the idea of boosting the immune response against the tumor and guaranteeing the accomplishment of CI blockade immunotherapy. These approaches included simple vaccine preparations consisting of specific peptides and proteins, as well as more complex strategies, such as engineered cellular vaccines, DC vaccines, and virus-vectored vaccines (28).

When CD103^+^ cDC1 were identified as the intratumoral population responsible for transporting and cross-presenting antigens and recruiting effector cells to the tumor site, the focus of investigations switched to finding new targets exclusively expressed on this population in order to increase these abilities with a consequential improved anti-tumor immune response. In general, the strategies reported so far have used costimulatory molecules agonists (i.e., CD40 antibodies) (29) or innate immune receptor agonists (Toll-like receptor ligands) (30) which strongly activate CD103^+^ cDC1 but also other cell populations, with consequently unpredicted risks. Recently, it was shown that neutralizing TIM-3, which is expressed on many cell types under homeostatic conditions but is restricted to CD103^+^ cDC1 inside the tumor, promotes CXCL9 expression by these cells and indirectly enhances the CD8^+^ T cell response in a breast cancer model (31). Moreover, expansion and activation of CD103^+^ cDC1 progenitors at the tumor site by simultaneously administering FLT3L and poly I:C enhances tumor responses to BRAF inhibitors and PD-L1 blockade (26).

By using a newly developed TLR3-GFP reporter mouse, we showed that inside B16-OVA tumors, cDCs are the main immune cells expressing TLR3. In our model, the totality of intratumoral CD103^+^ cDC1 express high levels of TLR3, whereas just a proportion of CD11b^+^ cDC2 do so and at lower levels. Interestingly, in draining lymph nodes only the CD103^+^ cDC1 express TLR3 while CD11b^+^ cDC2 present there did not, suggesting that TLR3 expression is induced in CD11b^+^ cDC2 inside the tumor by an unknown mechanism. Whereas, the global distribution of TLRs and other innate immune receptors is largely known (32) and the use of TLR agonists in cancer immunotherapy has been in the spotlight for many years, the fact that TLR3 is particularly found in CD103^+^ cDC1 inside the tumor, sets it apart from the rest of TLRs and turns it into an attractive target for immunotherapy.

Newly developed TLR3 agonists have been generated: ARNAX, for example, seems to engage only TLR3, and not the MAVS/RIG-like receptors, and it is also highly effective in combination with PD1/PDL1 blockade (33). Interestingly, ARNAX also licenses DCs to activate functional tumor-specific CTLs when administered in combination with tumor-associated antigens (33). Unlike poly I:C, poly A:U seems to be recognized only by TLR3 and no other cytosolic receptors, it was included in large clinical trials in the 80's showing promising results (12, 13, 34). More recent studies in gastric cancer patients revealed a beneficial outcome of this immunotherapy with no reported toxic side effects (35). In the present work, we have deepened our studies regarding the therapeutic efficacy of poly A:U and we exhaustively characterized the changes in the immune infiltrate after intratumoral poly A:U administration. Poly A:U treatment inhibits tumor growth and prolongs tumor-bearing mice survival in several murine models of cancer (15). This effect relies on IFNβ production in the tumor bed, readily visible a few hours after the first dose of poly A:U (15), which allows us to assume that CD103^+^ cDC1 and CD11b^+^ cDC2, are the initial source of IFNβ. This first encounter and its consequences profoundly shapes the immune infiltrate, enhancing the frequency of CD86^+^ cells among both cDC1 and cDC2 within the tumor. Interestingly, when studying these two cDCs subsets present in the tumor-draining lymph node, we detected a more activated phenotype only on CD103^+^ cDC1 and not on CD11b^+^ cDC2, indicating that either activated CD103^+^ cDC1 traffic to the lymph node or poly A:U reaches the lymph node activating the only TLR3-expressing population there, i.e., CD103^+^ cDC1.

Monocyte-derived cells and macrophages are the most abundant cells among tumor-infiltrating leukocytes. It has been proposed that all macrophages inside the tumor are derived from CD11b^+^Ly6C^hi^ monocytes, which proliferate and differentiate into the heterogeneous pool of tumor associated macrophages (TAM). Movahedi et al. (36) showed that the main type of monocytes recruited to the tumor are Ly6C^hi^ monocytes and downregulation of Ly6C would be the first step toward macrophage differentiation: monocytes would first give rise to Ly6C^int^ TAMs, which would then differentiate into MHCII^hi^ and MHCII^low^ TAMs. These latter populations present a highly differential gene expression profile that could easily be associated with a M1 or M2 polarization state. According to this, CD11b^+^Ly6C^hi^CCR2^+^ cells in our analysis could correlate with recently recruited monocytes that have not yet diminish too much their Ly6C expression but somehow are modulating their MHCII expression levels as they infiltrate the tumor. In our model, both MHCII^+^ and MHCII^−^ Ly6C^hi^CCR2^+^ cells are present approximately at the same number but the amount of these monocyte-derived cells producing TNF increases after the administration of poly A:U, in accordance with previous *in vitro* reports, that show that tumor-supporting myeloid cells can be converted to tumoricidal effectors by activating TLR3 (37). Moreover, we observed a decrease in the frequency and total number of M1-like and M2-like macrophages within the myeloid compartment, the latter being a key source of intratumoral IL10, indicating that poly A:U reduces the amount of this immunosuppressive cytokine present in the tumor (38, 39). Unexpectedly, cDCs also decrease in number and this does not seem to be a consequence of migration to lymph nodes. Rather, it could reflect their activation or a direct effect of the IFNβ elicited after TLR3 triggering. Indeed, it has been shown in different infection models that type I IFN elicited by the microorganism is responsible for macrophage and dendritic cell death (40–42).

Nonetheless, the most striking changes are seen within the lymphoid compartment, with all the anti-tumoral parameters being enhanced: a higher frequency of CD8^+^ Granzyme B^+^ T cells, (lower Treg/CD8^+^ ratio) and an important expansion of OVA-Tet^+^ CD8^+^ T cells. It is interesting to note that the mere administration of naked poly A:U alone was capable of expanding and activating tumor-specific clones. Interestingly, unsupervised analysis of the flow cytometry data indicate that granzyme B expression in the CD8^+^ T cell cluster, largely exceeds the area in which OVA-Tet^+^ CD8^+^ T cells clusters are found, indicating that the treatment expands and activates many more CD8^+^ T cell clones than the OVA-specific ones. This could be due to bystander activation of CD8^+^ T cells (43) or that clones specific for other tumor-antigens are being activated. Poly A:U also impacts profoundly on NK activation, which besides their well-known ability for killing cancer cells, can modulate the tumor microenvironment and act synergistically with CI blockade against the tumor (44, 45).

B cells are also drastically reduced in poly A:U -treated tumors. B cells have a controversial role in anti-tumor immunity with reports indicating an augmented T cell-mediated tumor response in genetically B cell-deficient mice (46, 47). On the other hand, many reports show that B cells enhance the anti-tumor activity of T cells (48, 49). Further studies should be done to understand if poly A:U treatment promotes a decrease in regulatory B cells, or conversely if it depletes the tumor site of effector B cells. Moreover, the role of B cells in B16 melanoma has been also a matter of discussion (50, 51) and so far, no clear data is available regarding the benefit of manipulating this population in solid tumors.

The PD1-PDL1 axis is also affected by poly A:U treatment; most OVA-Tet^+^ CD8^+^ T cells express PD1 accompanied by an increase in the percentage of total CD8^+^ T cells positive for this marker. Similarly, there is an increase in the frequency of PDL1^+^ cells among some myeloid populations, placing poly A:U as a great candidate to be used in combination with anti-PD1/PDL1 therapies. Therapeutic settings combining poly I:C with anti PD1/PDL1 have been assayed although it is well-known that poly I:C binds other cytosolic receptors (52, 53) and its use in clinical trials has been associated with many toxic side effects (54–56). Poly A:U can be proposed as an alternative adjuvant because of its accurate specificity for TLR3 shown by the lack of IFNβ response in UNC93B1 mutant mice and in TLR3^−/−^ mice (57). Intriguingly, the use of poly A:U in clinical trials has come to an impasse, and no clinical evaluation of its efficacy is being performed at the moment. Our results shed new light to promote further assays in this forgotten dsRNA mimetic to the clinical field.

## Data Availability

All datasets generated for this study are included in the manuscript and/or the supplementary files.

## Ethics Statement

Animal experimentation protocols were approved by Ethics Committees both at *Institut Curie* (NRC 2011) and *Centro de Investigaciones en Bioquimica Clinica e Inmunologia* (*CICUAL*). The mice were used in agreement with European and National regulations for the Protection of Vertebrate Animals used for Experimental and other Scientific Purposes. Care and use of animals also complied with internationally established principles of replacement, reduction, and refinement in accordance with the Guide for the Care and Use of Laboratory Animals (NRC 2011).

## Author Contributions

MM contributed with the conception and design of the study and wrote the first draft of the manuscript. ER, PA, and GG performed mice experiments, flow cytometry, and the statistical analysis. NN performed the unsupervised bioinformatics analysis (tSNE). FG and PB provided the TLR3-GFP mice and helped with the design of these experiments. CS and EP helped with the multiparametric flow cytometry and wrote sections of the manuscript. All authors contributed to manuscript revision, read, and approved the submitted version.

### Conflict of Interest Statement

The authors declare that the research was conducted in the absence of any commercial or financial relationships that could be construed as a potential conflict of interest.

## Supplementary Material

Supplemental Figure 1 *In vivo* and *in vitro* characterization of *Tlr3-EGFP* (B6*-Tlr3*^*tm*2*Ciphe*^) mice. **(A)** Mice homozygous for the *Tlr3-EGFP* allele (TLR3-KI^gfp/gfp^) together with mice heterozygous for this allele (TLR3-KI^gfp/wt^) and its wild-type control (TLR3-KI^wt/wt^) were intraperitoneally (i.p.) treated with either poly I:C (pIC-200 μg/mouse) or PBS as control, 24 h later the spleen was harvested and analyzed by flow cytometry for the expression of GFP. **(B)** Splenocytes from the three strains of mice were analyzed by flow cytometry showing the frequency of GFP^+^ cells among the different analyzed populations. **(C)** Dot plots from a representative animal from each strain of mice showing the frequency of GFP^+^ cells within total splenic DCs (CD11c^+^MHCII^+^), splenic CD8α^+^ DCs (CD11c^+^MHCII^+^CD8α^+^), and splenic CD8α^−^ DCs (CD11c^+^MHCII^+^CD8α^−^). **(D)** Expression level of CD86 (upper panel) and MHCII (lower panel) shown as MFI in splenic CD8α^+^ DCs both positive and negative for GFP isolated from the three strains of mice, treated with pIC or PBS as control. **(E)** Bone marrow-derived macrophages (BMDMs) from the three strains of TLR3-KI mice together with TLR3^KO^ mice were treated with poly A:U (pAU) at two concentrations (25 and 50 μg/mL) and pIC (50 μg/mL) for 24 h and analyzed for surface expression of CD80, CD86, and PDL1 by flow cytometry. Data is show as mean±SEM and each condition was statistically compared to control (RPMI) by two-way ANOVA. ^*^*p* < 0.05; ^**^*p* < 0.01; ^****^*p* < 0.0001.

Supplemental Figure 2 Side by side comparison of the frequencies of immune cell populations in spleens from wild type, homozygous (TLR3-KI^gfp/gfp^) and heterozygous TLR3-GFP reporter (TLR3-KI^gfp/wt^) mice. **(A)** Mice homozygous for the *Tlr3-EGFP* allele (TLR3-KI^gfp/gfp^) together with mice heterozygous for this allele (TLR3-KI^gfp/wt^) and its wild-type control (TLR3-KI^wt/wt^) were intraperitoneally (i.p.) treated with either poly I:C (pIC-200 μg/mouse) or PBS as control, 24 h later the spleen was harvested and analyzed by flow cytometry for the expression of T, B, myeloid, and dendritic cells. Results are expressed as percentages of CD45+ cells; each dot represents an animal.

Supplemental Figure 3 Characterization of tumor-infiltrating immune cells after poly A:U treatment. **(A)** Gating strategy used to characterize both myeloid and lymphoid cells infiltrating B16-OVA tumors. **(B)** Representative histogram showing the expression of different surface markers on tumor-infiltrating myeloid cells from a control animal (PBS) shaded in gray together with the respective isotype control. *Ex vivo* analyses were performed at day 13 post-tumor inoculation from WT C57BL/6 mice.

Supplemental Figure 4 Frequencies of tumor-infiltrating immune populations after administration of poly A:U. **(A)** Frequency among CD45^+^ cells of the different myeloid cells infiltrating poly A:U-treated (pAU) and non-treated (PBS) B16-OVA tumors. **(B)** Frequency among CD45^+^ cells of the different lymphoid cells infiltrating poly A:U-treated (pAU) and non-treated (PBS) B16-OVA tumors. **(C)** Frequency among CD45^+^ cells of the different immune populations infiltrating poly A:U-treated (pAU) and non-treated (PBS) B16-OVA tumors. *Ex vivo* analyses were performed at day 13 post-tumor inoculation from WT C57BL/6 mice. ^*^*p* < 0.05; ^**^*p* < 0.01; ^***^*p* < 0.001; ^****^*p* < 0.0001.

Supplemental Figure 5 tSNE analysis objectively delineates the different immune cell subsets present within B16-OVA tumor. **(A)** tSNE dimensionality reduction showing concatenated flow cytometry data of intratumoral immune cells from mice treated with PBS (control) or poly A:U (pAU) with heat-map showing the distribution of various surface markers on the different clusters. **(B)** Frequency of the different tumor-infiltrating immune cells obtained by FlowSOM clustering on each individual mouse. Box and whiskers plots showing frequencies of the different populations in PBS (control) or poly A:U treated animals. **(C)** Heat-map showing the MFI for the specified markers on the different tumor-infiltrating immune cells from the control (PBS) mice obtained by an unsupervised analysis. *Ex vivo* analyses were performed at day 13 post-tumor inoculation from WT C57BL/6 mice.

Supplemental Table 1 Antibodies used for flow cytometry analysis.
