# Supplementary figures and images for: TLR3 Activation of Intratumoral CD103+ Dendritic Cells Modifies the Tumor Infiltrate Conferring Anti-tumor Immunity
Source: Front Immunol. 2019 Mar 20;10:503. doi: 10.3389/fimmu.2019.00503 (PMC6435583; doi:10.3389/fimmu.2019.00503)

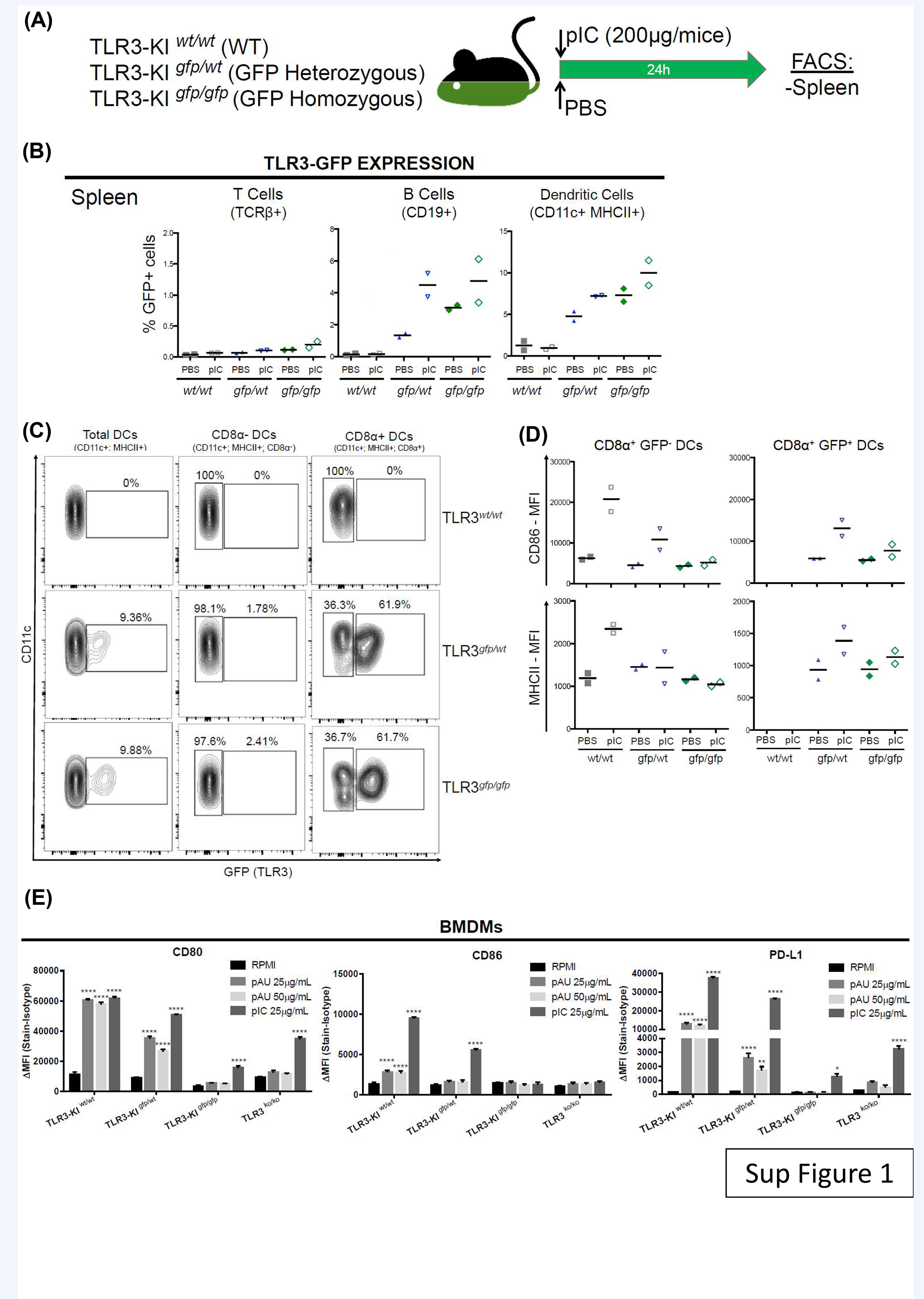

Supplement: Supplemental Figure 1 — In vivo and in vitro characterization of Tlr3-EGFP (B6-Tlr3tm2Ciphe) mice. (A) Mice homozygous for the Tlr3-EGFP allele (TLR3-KIgfp/gfp) together with mice heterozygous for this allele (TLR3-KIgfp/wt) and its wild-type control (TLR3-KIwt/wt) were intraperitoneally (i.p.) treated with either poly I:C (pIC-200 μg/mouse) or PBS as control, 24 h later the spleen was harvested and analyzed by flow cytometry for the expression of GFP. (B) Splenocytes from the three strains of mice were analyzed by flow cytometry showing the frequency of GFP+ cells among the different analyzed populations. (C) Dot plots from a representative animal from each strain of mice showing the frequency of GFP+ cells within total splenic DCs (CD11c+MHCII+), splenic CD8α+ DCs (CD11c+MHCII+CD8α+), and splenic CD8α− DCs (CD11c+MHCII+CD8α−). (D) Expression level of CD86 (upper panel) and MHCII (lower panel) shown as MFI in splenic CD8α+ DCs both positive and negative for GFP isolated from the three strains of mice, treated with pIC or PBS as control. (E) Bone marrow-derived macrophages (BMDMs) from the three strains of TLR3-KI mice together with TLR3KO mice were treated with poly A:U (pAU) at two concentrations (25 and 50 μg/mL) and pIC (50 μg/mL) for 24 h and analyzed for surface expression of CD80, CD86, and PDL1 by flow cytometry. Data is show as mean±SEM and each condition was statistically compared to control (RPMI) by two-way ANOVA. *p < 0.05; **p < 0.01; ****p < 0.0001. [file Image_1.TIF]

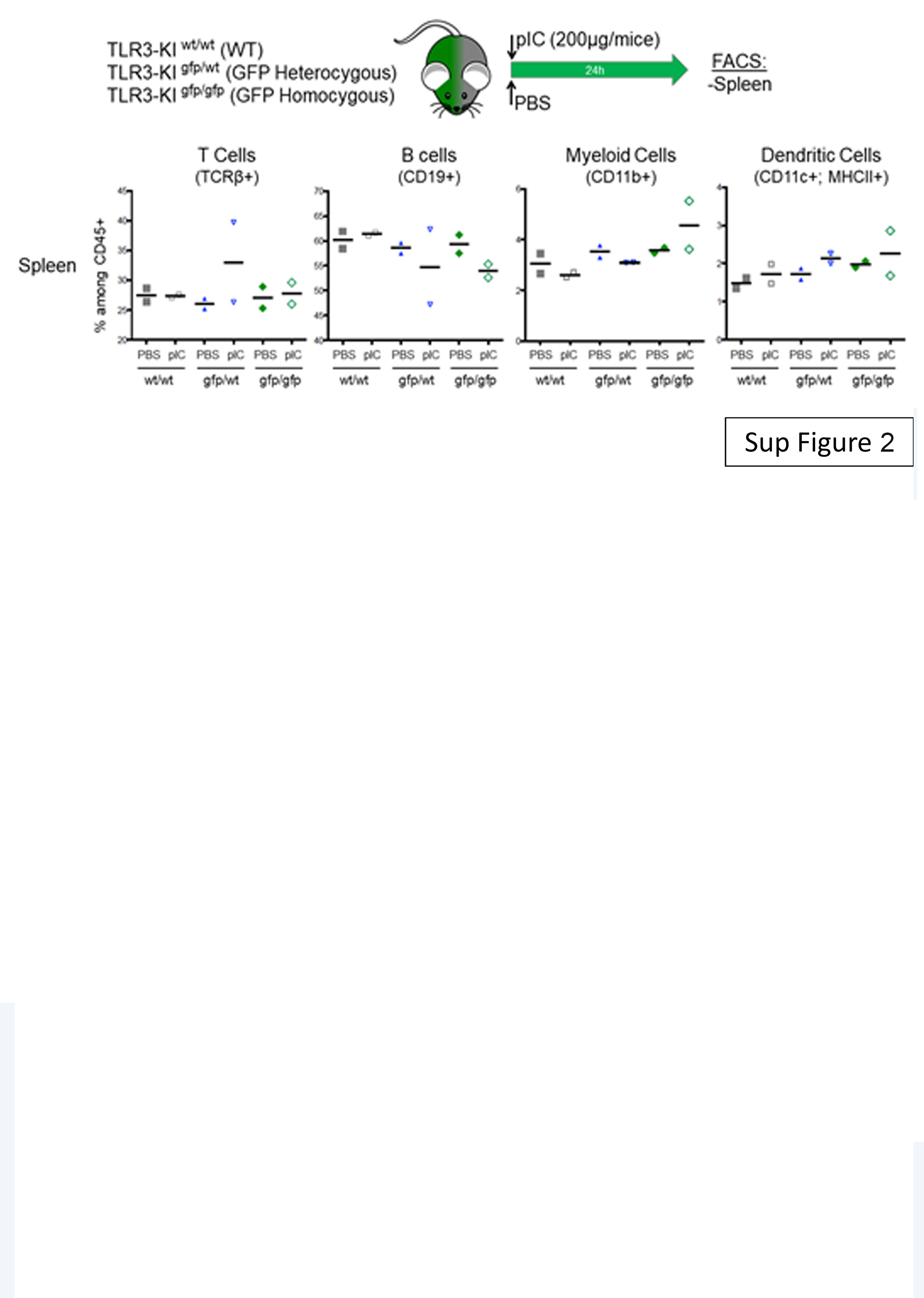

Supplement: Supplemental Figure 2 — Side by side comparison of the frequencies of immune cell populations in spleens from wild type, homozygous (TLR3-KIgfp/gfp) and heterozygous TLR3-GFP reporter (TLR3-KIgfp/wt) mice. (A) Mice homozygous for the Tlr3-EGFP allele (TLR3-KIgfp/gfp) together with mice heterozygous for this allele (TLR3-KIgfp/wt) and its wild-type control (TLR3-KIwt/wt) were intraperitoneally (i.p.) treated with either poly I:C (pIC-200 μg/mouse) or PBS as control, 24 h later the spleen was harvested and analyzed by flow cytometry for the expression of T, B, myeloid, and dendritic cells. Results are expressed as percentages of CD45+ cells; each dot represents an animal. [file Image_2.TIF]

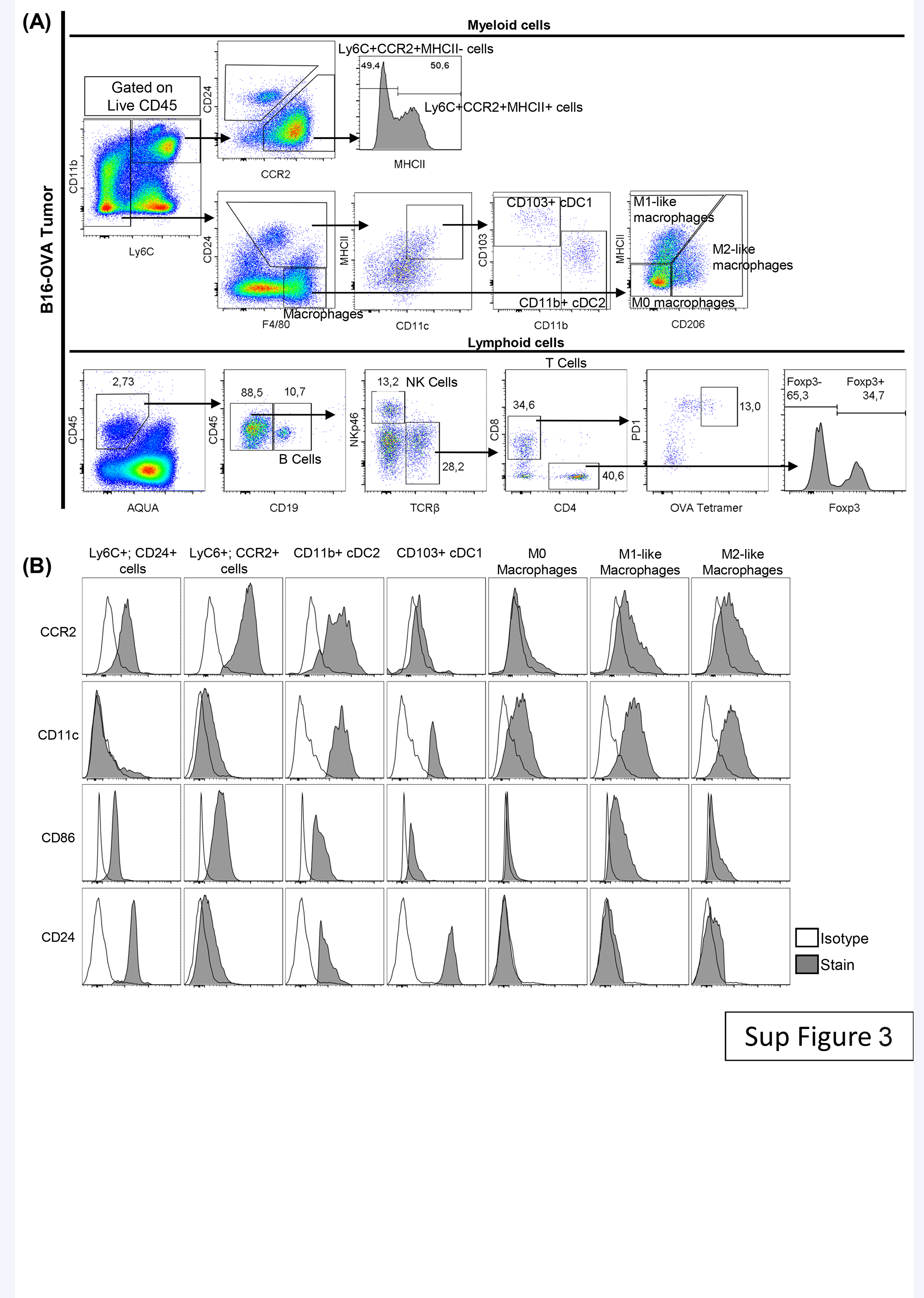

Supplement: Supplemental Figure 3 — Characterization of tumor-infiltrating immune cells after poly A:U treatment. (A) Gating strategy used to characterize both myeloid and lymphoid cells infiltrating B16-OVA tumors. (B) Representative histogram showing the expression of different surface markers on tumor-infiltrating myeloid cells from a control animal (PBS) shaded in gray together with the respective isotype control. Ex vivo analyses were performed at day 13 post-tumor inoculation from WT C57BL/6 mice. [file Image_3.TIF]

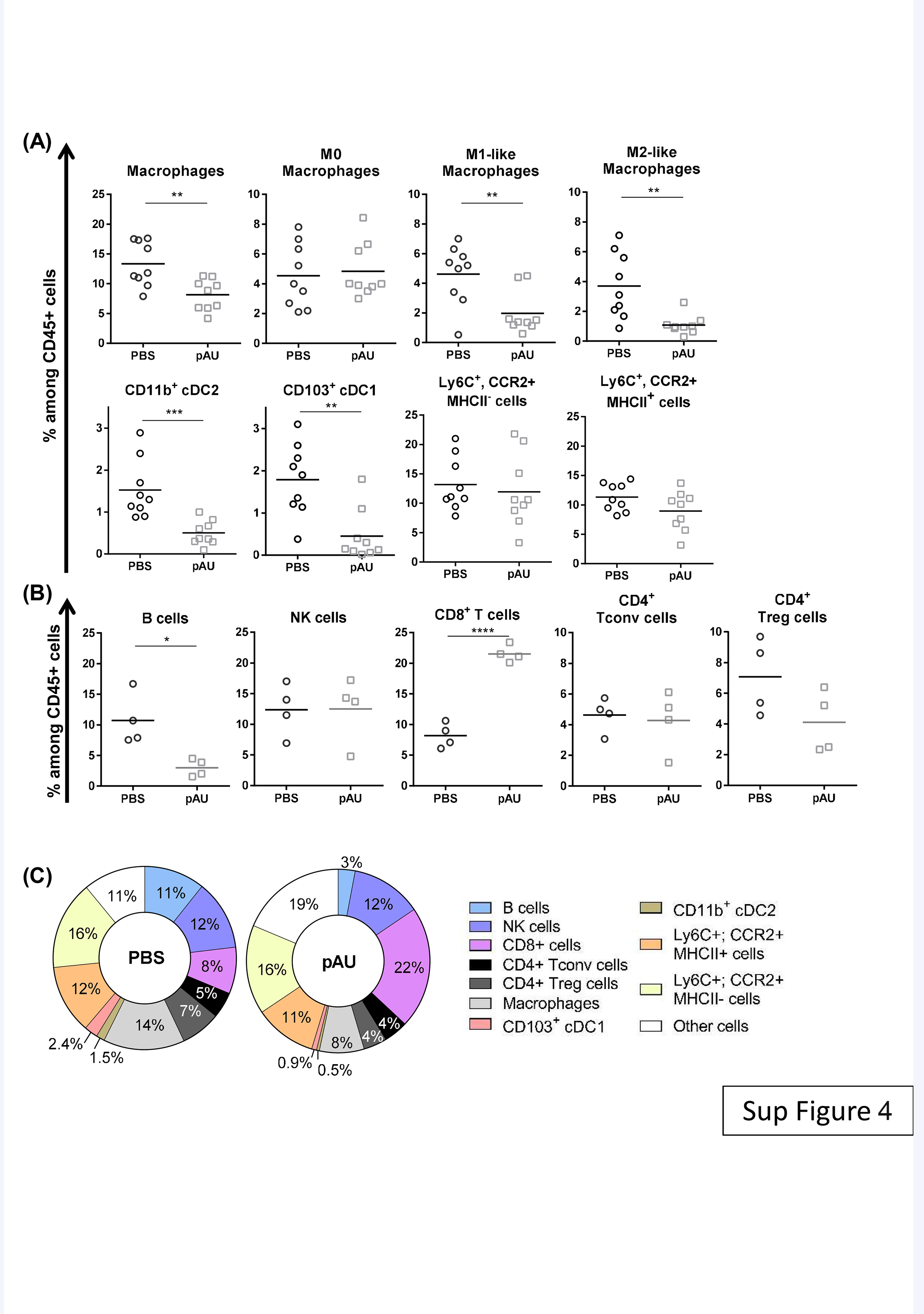

Supplement: Supplemental Figure 4 — Frequencies of tumor-infiltrating immune populations after administration of poly A:U. (A) Frequency among CD45+ cells of the different myeloid cells infiltrating poly A:U-treated (pAU) and non-treated (PBS) B16-OVA tumors. (B) Frequency among CD45+ cells of the different lymphoid cells infiltrating poly A:U-treated (pAU) and non-treated (PBS) B16-OVA tumors. (C) Frequency among CD45+ cells of the different immune populations infiltrating poly A:U-treated (pAU) and non-treated (PBS) B16-OVA tumors. Ex vivo analyses were performed at day 13 post-tumor inoculation from WT C57BL/6 mice. *p < 0.05; **p < 0.01; ***p < 0.001; ****p < 0.0001. [file Image_4.TIF]

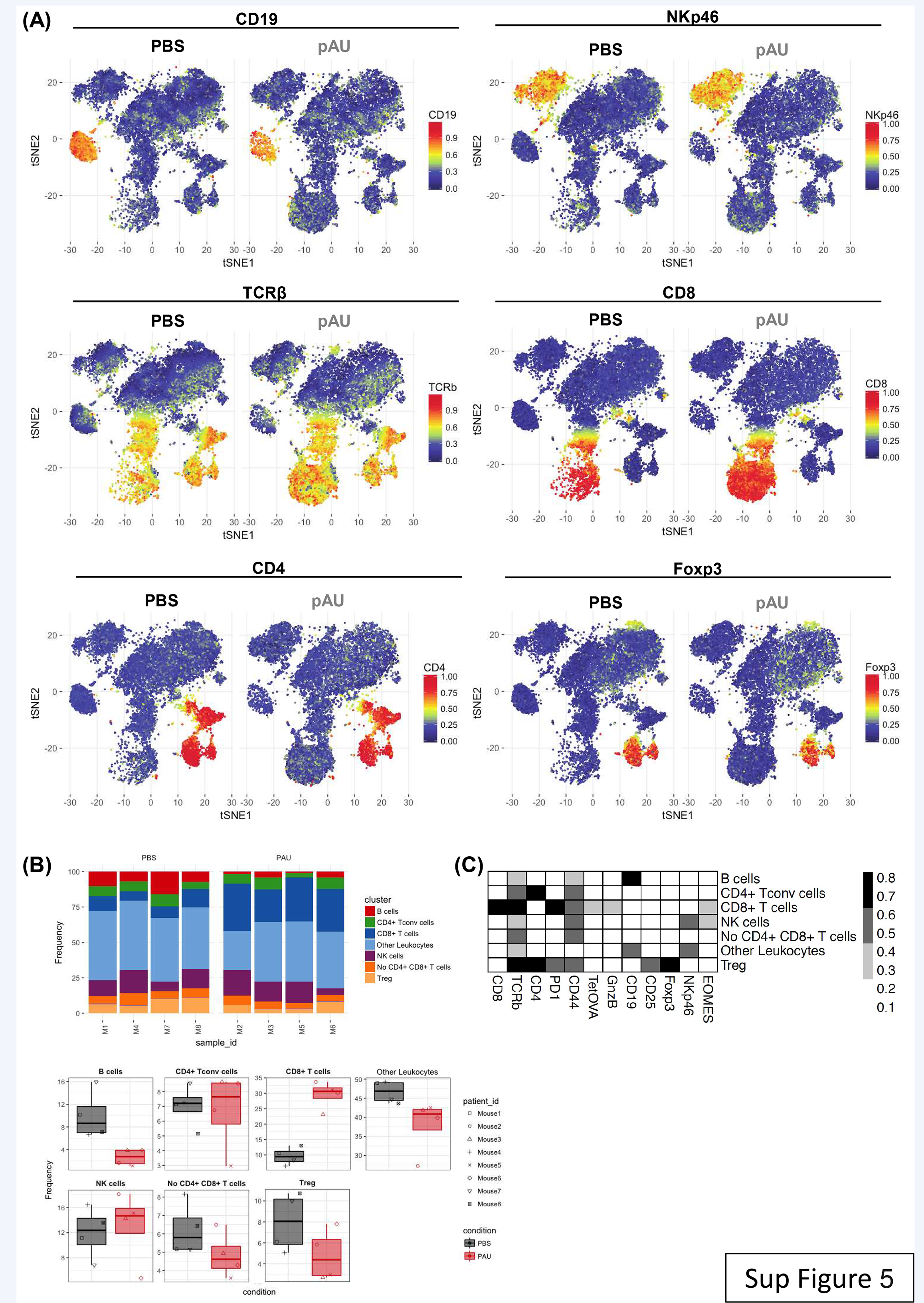

Supplement: Supplemental Figure 5 — tSNE analysis objectively delineates the different immune cell subsets present within B16-OVA tumor. (A) tSNE dimensionality reduction showing concatenated flow cytometry data of intratumoral immune cells from mice treated with PBS (control) or poly A:U (pAU) with heat-map showing the distribution of various surface markers on the different clusters. (B) Frequency of the different tumor-infiltrating immune cells obtained by FlowSOM clustering on each individual mouse. Box and whiskers plots showing frequencies of the different populations in PBS (control) or poly A:U treated animals. (C) Heat-map showing the MFI for the specified markers on the different tumor-infiltrating immune cells from the control (PBS) mice obtained by an unsupervised analysis. Ex vivo analyses were performed at day 13 post-tumor inoculation from WT C57BL/6 mice. [file Image_5.TIF]
