# Supplementary material for: TLR3 Activation of Intratumoral CD103+ Dendritic Cells Modifies the Tumor Infiltrate Conferring Anti-tumor Immunity
Source: Front Immunol. 2019 Mar 20;10:503. doi: 10.3389/fimmu.2019.00503 (PMC6435583; doi:10.3389/fimmu.2019.00503)
Supplement: Supplemental Table 1 — Antibodies used for flow cytometry analysis. [file Table_1.pdf]

Supplemental Table 1: Antibodies used for flow cytometry analysis.

REAGENT	FLUOROCHROME	SOURCE	IDENTIFIER
CD45.2	APC-Cy7	BioLegend	109824
CD45.2	Alexa Fluor 700	BioLegend	109822
CD103	PerCP-eF710	eBioscience	46-1031-82
MHCII	Alexa Fluor 700	eBioscience	56-5321-82
F4/80	BV605	BioLegend	123133
Ly6C	BV785	BioLegend	128041
CD8α	BUV737	BD Biosciences	564297
CD8α	BUV395	BD Biosciences	565968
CD206	APC	BioLegend	141708
CD86	BUV395	BD Biosciences	564199
CD24	PE-Cy5	eBioscience	15-0242-81
CD11c	PE-Texas Red	Thermo Fischer Scientific	MCD11C17
CCR2	BV421	BioLegend	150605
CD11b	FITC	BioLegend	553310
CD19	PE-Cy7	BioLegend	115520
TCRβ	PE-Cy7	BD Biosciences	560729
TCRβ	APC-Cy7	BioLegend	109220
NKp46	PE-Cy7	BioLegend	137617
PD-L1	PE	eBioscience	12-5982-81
PD1	BV785	BioLegend	135225
Foxp3	eFluor 450	Thermo Fischer Scientific	48-5773-82
KLRG1	Alexa Fluor 488	BD Biosciences	561619
IL10	PE-Cy7	BioLegend	505025
CD25	PE-CF594	BD Biosciences	562694
iTAg Tetramer - H-2 Kb OVA (SIINFEKL)	PE	MBL Int. Corp.	TB-5001-1
Granzyme B	Alexa Fluor 647	BioLegend	515405
CD4	BUV737	BD Biosciences	564933
LIVE/DEAD Fixable Aqua Dead Cell Stain	Ex: 405nm - Em: 525nm	Thermo Fischer Scientific	L34957
